# Whole-Genome Sequence of High-Risk Clone Sequence Type 111 of Pseudomonas aeruginosa Strain NUBRI-P, Isolated from a Wounded Sudanese Patient

**DOI:** 10.1128/MRA.00879-19

**Published:** 2019-10-03

**Authors:** Sofia B. Mohamed, Sumaya Kambal, Abdalla Munir, Nusiba I. Abdalla, Ahmed Hamad, Sara E. Mohammed, Fatima E. Ahmed, Omnia Hamid, Mohamed M. Hassan, Arshad Ismail, Mushal Allam

**Affiliations:** a Bioinformatics and Biostatistics Department, National University Biomedical Research Institute, National University, Khartoum, Sudan; b Sequencing Core Facility, National Institute for Communicable Diseases, National Health Laboratory Service, Johannesburg, South Africa; University of Maryland School of Medicine

## Abstract

Pseudomonas aeruginosa is a common nosocomial pathogen often associated with a high mortality rate in vulnerable populations. Here, we describe the genomic sequence of a pan-resistant, high-risk clone of P. aeruginosa sequence type 111 (ST111) isolated from a hospital patient in Sudan.

## ANNOUNCEMENT

Pseudomonas aeruginosa is an opportunistic Gram-negative pathogen noted for its intrinsic resistance to multiple antibiotics. Due to increasing multidrug resistance, treatment options for P. aeruginosa infections are limited, and treatment requires new therapeutic approaches (1, 2). Here, we report the draft genome sequence of a pan-resistant, high-risk clone of a P. aeruginosa strain (NUBRI-P) isolated from Sudan.

P. aeruginosa strain NUBRI-P was isolated from a postoperative wound infection of a 52-year-old male admitted to the Khartoum North Teaching Hospital in Sudan. The specimen was directly inoculated on MacConkey agar and then was incubated overnight under aerobic conditions at 37°C. The colony was identified using Gram stain and biochemical properties assessed with an oxidase test, a catalase test, Kligler’s iron agar (KIA), sulfide indole motility (SIM), a citrate agar test, and a urea test (3). The analytical profile index (API20-NE) was used to confirm the isolate. Disc diffusion testing was carried out on the isolate according to Clinical and Laboratory Standards Institute (CLSI M100 2007) guidelines (4). The isolate showed resistance to 18 of the antibiotics tested, namely, imipenem (10 μg), meropenem (10 μg), ertapenem (10 μg), amikacin (30 μg), co-trimoxazole (23.75 μg), ampicillin (10 μg), aztreonam (15 μg), amoxiclav (30 μg), ceftazidime (30 μg), ceftriaxone (30 μg), cefotaxime (10 μg), ciprofloxacin (5 μg), tetracycline (30 μg), norfloxacin (10 μg), naldixic acid (30 μg), gentamicin (10 μg), tobramycin (10 μg), and colistin (10 μg). The strain was subcultured 10 times before the DNA extraction. The genomic DNA was extracted from three colonies using a QIAamp DNA minikit (Qiagen, Germany). Paired-end libraries were prepared using the Nextera DNA Flex library prep kit, followed by 2 × 300-bp sequencing on a MiSeq platform (Illumina, Inc., USA). The resultant paired-end reads were quality trimmed using Sickle version 1.33 (5) and *de novo* assembled using SPAdes version 3.11 (6) with default settings. The contiguous sequences were then submitted to the NCBI Prokaryotic Genome Annotation Pipeline (7). Multilocus sequence typing and the prediction of resistance genes were performed using GoSeqIt (https://www.goseqit.com/) (8). The Research Ethics Committee at the National University in Sudan approved this study (NU-REC90).

A total of 1,351,018 paired-end reads were obtained from the whole-genome sequence of NUBRI-P. Quality-controlled reads (1,348,461 reads; average length, 267.7 bp; 56-fold read depth of the genome) with a Phred score of >20 were assembled *de novo* with a minimum contig cutoff of 200 bp for 99 contigs (average contig length, 65,172 bp; *N*_50_, 219,079 bp). The total genome length was 6,452,072 bp with a GC content of 66.3%. In total, the NUBRI-P genome contains 6,247 genes (6,183 protein-coding genes and 64 RNA genes). The multilocus sequence type (MLST) was deﬁned as sequence type 111 (ST111). According to the P. aeruginosa PubMLST database (https://pubmlst.org/paeruginosa/), ST111 is reported mainly from Europe, North and South America, and Asia. By using the ResFinder database within the GoSeqIt pipeline, five antibiotic resistance genes were found in NUBRI-P, including the aminoglycoside resistance gene *aph(3′)-IIb* (GenBank accession no. TGO97691), the beta-lactamase resistance gene *bla*_OXA-395_ (TGO99202), the fluoroquinolone resistance gene *crpP* (TGP00565), and the fosfomycin resistance gene *murA* (TGP01273).

This whole-genome sequence of the globally dominant high-risk clone ST111 of P. aeruginosa, isolated from Sudan, and its resistance markers have the potential to improve our understanding of various molecular mechanisms, including antibiotic resistance and pathogenicity.

### Data availability.

This whole-genome shotgun project has been deposited in DDBJ/ENA/GenBank under the accession no. SMZF00000000. The version described in this paper is the first version, SMZF01000000. The raw sequencing reads have been submitted to the SRA under the accession no. SRR8729748.
